# Identifying hub genes and miRNAs in Crohn’s disease by bioinformatics analysis

**DOI:** 10.3389/fgene.2022.950136

**Published:** 2022-08-31

**Authors:** Yuxin Sun, Daxing Cai, Weitao Hu, Taiyong Fang

**Affiliations:** ^1^ Department of Gastroenterology, The Second Affiliated Hospital of Fujian Medical University, Quanzhou, Fujian, China; ^2^ Department of Rheumatology, The Second Affiliated Hospital of Fujian Medical University, Quanzhou, Fujian, China

**Keywords:** Crohn’s disease, bioinformatics analysis, differentially expressed genes, MicroRNAs, hub genes

## Abstract

**Introduction:** Crohn’s disease (CD) is a disease that manifests mainly as chronic inflammation of the gastrointestinal tract, which is still not well understood in terms of its pathogenesis. The aim of this study was to use bioinformatics analysis to identify differentially expressed genes (DEGs) and miRNAs with diagnostic and therapeutic potential in CD.

**Materials and methods:** Three CD datasets (GSE179285, GSE102133, GSE75214) were downloaded from the Gene Expression Omnibus (GEO) database. DEGs between normal and CD tissues were identified using the GEO2R online tool. The Gene Ontology (GO) term and Kyoto Encyclopedia of Genes and Genomes (KEGG) pathway enrichment analyses of the DEGs were conducted using the clusterProfiler function in the R package. Protein-protein interaction network (PPI) analysis and visualization were performed with STRING and Cytoscape. Ten hub genes were identified using cytoHubba’s MCC algorithm and validated with datasets GSE6731 and GSE52746. Finally, the miRNA gene regulatory network was constructed by Cytoscape and NetworkAnalyst to predict potential microRNAs (miRNAs) associated with DEGs.

**Results:** A total of 97 DEGs were identified, consisting of 88 downregulated genes and 9 upregulated genes. The enriched functions and pathways of the DEGs include immune system process, response to stress, response to cytokine and extracellular region. KEGG pathway analysis indicates that the genes were significantly enriched in Cytokine-cytokine receptor interaction, IL-17 signaling pathway, Rheumatoid arthritis and TNF signaling pathway. In combination with the results of the protein-protein interaction (PPI) network and CytoHubba, 10 hub genes including IL1B, CXCL8, CXCL10, CXCL1, CXCL2, CXCL5, ICAM1, IL1RN, TIMP1 and MMP3 were selected. Based on the DEG-miRNAs network construction, 5 miRNAs including hsa-mir-21-5p, hsa-mir-93-5p, hsa-mir-98-5p, hsa-mir-1-3p and hsa-mir-335-5p were identified as potential critical miRNAs.

**Conclusion:** In conclusion, a total of 97 DEGs, 10 hub genes and 5 miRNAs that may be involved in the progression or occurrence of CD were identified in this study, which could be regarded as biomarkers of CD.

## Introduction

Crohn’s disease (CD) is one of the inflammatory bowel diseases (IBD), mainly manifesting as chronic inflammation of different parts of the gastrointestinal tract, with a progressive and destructive course, whose incidence has been increasing in recent years (Roda et al., 2020). CD is still unclear in its etiology, but genetic, immune, and environmental factors increase its risk of development and progression (Torres et al., 2017). Crohn’s disease shows an overlap with regard to disease behaviour with ulcerative colitis (Atreya and Siegmund, 2021). The course of CD is progressive and destructive, and systemic and extra-intestinal manifestations can occur, which can seriously affect the quality of life and prognosis in patients (Ananthakrishnan et al., 2018).

Currently, promoting mucosal healing is the preferred treatment aim for CD (Bernstein et al., 2019). The use of anti-inflammatory treatments such as infliximab, adalimumab and Vedolizumab, for example, has transformed the management of CD in the last 2 decades (Dulai et al., 2016; Feagan et al., 2016). Although these targeted biologic therapies represent a significant advance in the treatment of CD, there are still some patients who are not sensitive to the targeted drugs (anti-TNF antibodies such as infliximab and adalimumab) that have been identified (Schmitt et al., 2021). However, biomarkers may help clinicians characterize disease severity and prognosis in early diagnosis and intervention, whereas biomarkers may be useful in defining treatment response and predicting postoperative CD recurrence. Therefore, the research and discovery of the precise molecular mechanisms of the disease are essential for the development of therapeutic strategies for CD.

Bioinformatics is an emerging subject that is already widely used for early diagnosis and predicting the prognosis of cancer patients (Wang and Liotta, 2011). This new approach has been used broadly in the study of various cancers (Li et al., 2017; Yan et al., 2018; Tsai and Gamblin, 2019), and has also played a role in the identification of a few new biomarkers for non-oncology diseases (Chen et al., 2018; Cakmak and Demir, 2020; Xie et al., 2020). Microarray technology is widely used to screen for genomic level differential alterations and can be used to participate in the prediction of CD development and progression. Nie et al. identified TLR2, TREM1, CXCR1, FPR1, and FPR2 as promising candidates for predicting anti-TNFα responses in CD patients by microarray analysis (Nie et al., 2022). Hu et al. found that Hsa_circ_0062142 and hsa_circ_0001666 may play a key role in pathogenesis and serve as potential biomarkers of CD by microarray analysis (Hu et al., 2021). MicroRNAs (miRNAs) are 19–25 nucleotide single-stranded non-coding RNA molecules which can inhibit translation and destabilize messenger RNAs (mRNAs). MiRNAs regulate gene expression by binding to mRNAs and may play a critical modulatory function in the progression of CD (Kalla et al., 2015). Growing evidence suggests that miRNAs contribute significantly to the complicated etiology and pathogenesis in CD (Schaefer et al., 2015). However, reliable results from individual microarray analysis are difficult to obtain owing to its high false positive rate. Accordingly, in our study, we downloaded 3 mRNA microarray datasets from Gene Expression Omnibus (GEO) and performed them to identify DEGs between normal and CD intestinal mucosal tissues. Afterwards, enrichment analysis of GO terms and KEGG pathways and PPI network analysis were conducted to identify the underlying molecular mechanisms of CD onset and progression. Lastly, miRNA gene regulatory networks were construct for predicting potential microRNAs (miRNAs) associated with DEGs with the use of Cytoscape and NetworkAnalyst. In summary, there were 97 DEGs, 10 hub genes and 5 potential miRNAs that were identified as potential target biomarkers for CD.

## Materials and methods

### Microarray data

GEO (http://www.ncbi.nlm.nih.gov/geo) (Edgar et al., 2002) is a public functional genomics data repository of high throughput gene expression data, chips and microarrays. The GSE75214 (Vancamelbeke et al., 2017) and GSE102133 (Verstockt et al., 2019) datasets generated using the Affymetrix GPL6244 platform, (Affymetrix Human Genome 1.0 ST Array), and GSE179285 (Keir et al., 2021) generated on the GPL6480 platform (Agilent-014850 Whole Human Genome Microarray 4 × 44K G4112F) were downloaded from GEO. Annotated information from the platform was used to convert the probes to the corresponding gene symbols. The GSE179285 dataset contained 47 CD intestinal mucosa tissue samples and 31 controls; the GSE75214 dataset contained 59 CD samples and 22 healthy controls; and the GSE102133 dataset contained 65 intestinal mucosal biopsies from CD patients and 12 intestinal mucosal tissues from controls.

### Identification of DEGs

Identification of DEGs between CD and normal samples was performed using GEO2R (http://www.ncbi.nlm.nih.gov/geo/geo2r). GEO2R is an online interactive tool that allows users to identify DEGs for different experimental conditions by comparing two datasets in the GEO series (Barrett et al., 2013). Adjusted *p*-values (adj. P) and Benjamini and Hochberg’s false discovery rates were applied to provide a balance between discovering statistically significant genes and limiting false positives. Probe sets without corresponding gene symbols or genes with more than one probe set were deleted or normalized, respectively. |Log FC (fold change)| >1 and adj. *p*-value <0.01 were considered statistically significant.

### Enrichment analysis of KEGG and GO for DEGs

KEGG is a database resource for elucidating high-level functions and effects of biological systems (Kanehisa, 2002; Kanehisa et al., 2017). GO is a major bioinformatics initiative for high-quality functional gene annotation based on biological processes (BP), molecular functions (MF) and cellular components (CC) (Pomaznoy et al., 2018). GO term and KEGG pathway analyses were conducted using the clusterProfiler function in the R package. The cutoff criteria of *p* < 0.05 and FDR <0.05 were defined as significant.

### Construction of PPI network and module analysis

The PPI network was constructed using the Search Tool for the Retrieval of Interacting Genes (STRING; http://string-db.org) (version 11.5) (Franceschini et al., 2013) online database. Cytoscape (version 3.9.1) is an open-source bioinformatics software platform for visualizing molecular interaction networks (Smoot et al., 2011). Molecular Complex Detection (MCODE) (version 2.0) is a plug-in in Cytoscape used to identify densely connected regions by clustering a given network based on the topology (Bandettini et al., 2012). Using Cytoscape to map the PPI network, the MCODE was used to identify the most significant modules in the PPI network. The following selection criteria were used: MCODE scores >5, degree cut-off = 2, node score cut-off = 0.2, Max depth = 100 and k-score = 2.

### Selection and analysis of hub genes

The top 10 genes were obtained using MCC algorithm with Cytoscape’s plug-in cytoHubba. GO term and KEGG pathway analyses were conducted using the clusterProfiler function in the R package.

### Validation of hub gene expression of CD datasets

The two microarray datasets of CD (GSE6731: 7 inflamed CD vs. 4 healthy controls; GSE52746:10 active CD vs. 17 healthy controls) that were retrieved from the GEO database were used to verify the expressions of the hub genes The “limma” package was also applied to identify the DEGs with thresholds of |log2FC| ≥ 1 and adjust. *p* < 0.05. The results were visualized in volcano plots and the hub genes were marked.

### MiRNAs related to hub genes

The top 9 hub genes were mapped to the respective miRNAs with NetworkAnalyst 3.0 (Zhou et al., 2019) (https://www.networkanalyst.ca/), an online platform for visualization that helps to identify miRNA-gene interactions in Gene Regulatory Networks. For each hub gene, miRNAs were identified as having a degree cutoff = 1.0. Lastly, a mapping of these hub genes and miRNAs was performed by Cytoscape 3.9.1.

## Results

### Identification of DEGs in CD

**FIGURE 1:**
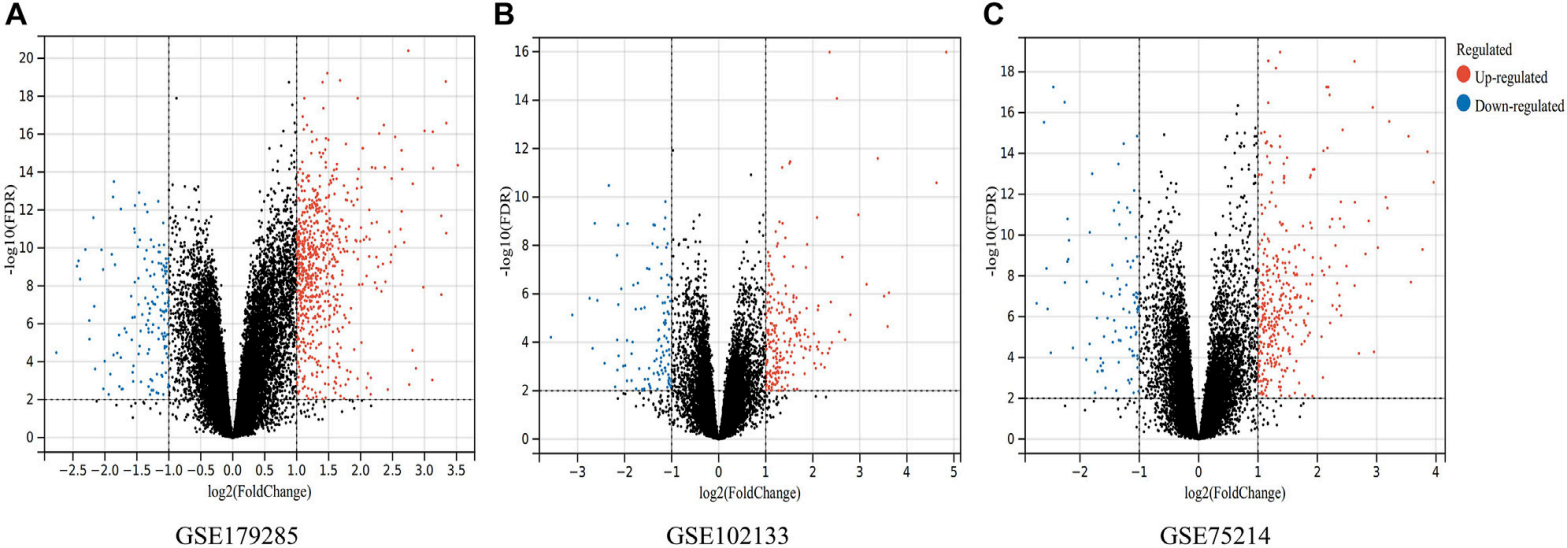
Volcano plots indicating differentially expressed genes (DEGs) among the control and CD groups. **(A–C)** DEGs of the GSE179285, GSE102133 and GSE75214 datasets are shown, separately. Red data points represent upregulated genes and blue ones represent downregulated genes. Genes without any significant differences are in black.

A total of three datasets (GSE179285, GSE75214 and GSE102133) containing gene expression profiles of both healthy and CD-active intestinal mucosal tissue samples were obtained from the GEO database. Details for the three datasets are shown in Table 1. DEGs were identified after normalization of microarray results (634 in GSE179285, 388 in GSE75214, and 291 in GSE102133). A total of 517 upregulated and 117 downregulated genes, 303 upregulated and 85 downregulated genes and 191 upregulated and 100 downregulated genes were included in the DEGs in the GSE179285, GSE75214 and GSE102133 datasets, respectively. All DEGs were identified by comparison of the gene expression profiles of normal healthy controls and CD samples. Figure 1 shows the gene expression profiles of DEGs in three datasets containing data from 2 sets of samples.

**TABLE 1:** Details for GEO Crohn’s disease data.

References	GEO	Platform	Control	CD
Verstockt S (2019)	GSE102133	GPL6244	12	65
Vancamelbeke M (2017)	GSE75214		22	59
Keir ME (2021)	GSE179285	GPL6480	31	47

**FIGURE 2:**
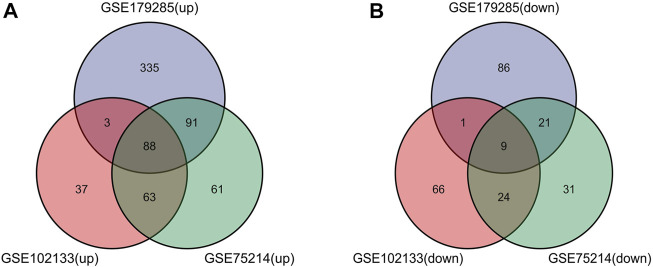
Venn diagrams showing the differentially expressed genes (DEGs) that overlapped among the 3 datasets retrieved from Gene Expression Omnibus (GEO). **(A,B)** Indicate the overlap of upregulated and downregulated genes in the GSE179285, GSE102133 and GSE75214 datasets, separately.

Such genes were presented by further screening and Venn diagrams were drawn to demonstrate these genes. The 97 DEGs were found to be significantly differentially expressed in the 3 groups, as shown in Figure 2, with 88 genes upregulated and 9 genes downregulated (Table 2).

**TABLE 2:** Screening DEGs in Crohn’s disease patients by integrated microarray.

DEGs	Gene terms
Upregulated	ADGRE2 ADGRG6 ANGPTL2 ANXA1 AQP9 BACE2 C2 CD274 CD55 CDH11 CDH3 CFB CFI CHI3L1 COL4A1 COL6A3 CTSK *CXCL1 CXCL10* CXCL11 *CXCL2 CXCL5 CXCL8* CXCL9 CXCR2 DMBT1 DRAM1 DUOX2 DUOXA2 FCGR3A FPR1 FPR2 FSTL1 GBP4 GBP5 HCAR3 *ICAM1* IDO1 IFITM1 IFITM3 IGFBP5 IGHV3-69–1///IGHV3OR16-7 IGKC *IL1B IL1RN* KCNE3 KYNU LAMP3 LCN2 LPL LUM MMP1 MMP10 MMP12 *MMP3* MUC1 MXRA5 NCF2 NOS2 PDZK1IP1 PLA2G7 PLAU RAB31 REG1A REG1B S100A8 S100P SAA2 SAMD9L SELP SERPINA3 SLAMF7 SLC6A14 SOCS3 SOD2 STAT1 TCN1 TFF1 TFPI2 *TIMP1* TMPRSS3 TNFAIP6 TNFSF13B TREM1 UBD VWF WARS WNT5A
Downregulated	ACSF2 CDHR1 CLDN8 GUCA2A MT1M PADI2 PAQR5 SLC26A2 TRPM6

### Enrichment analysis of KEGG and GO for DEGs

**FIGURE 3:**
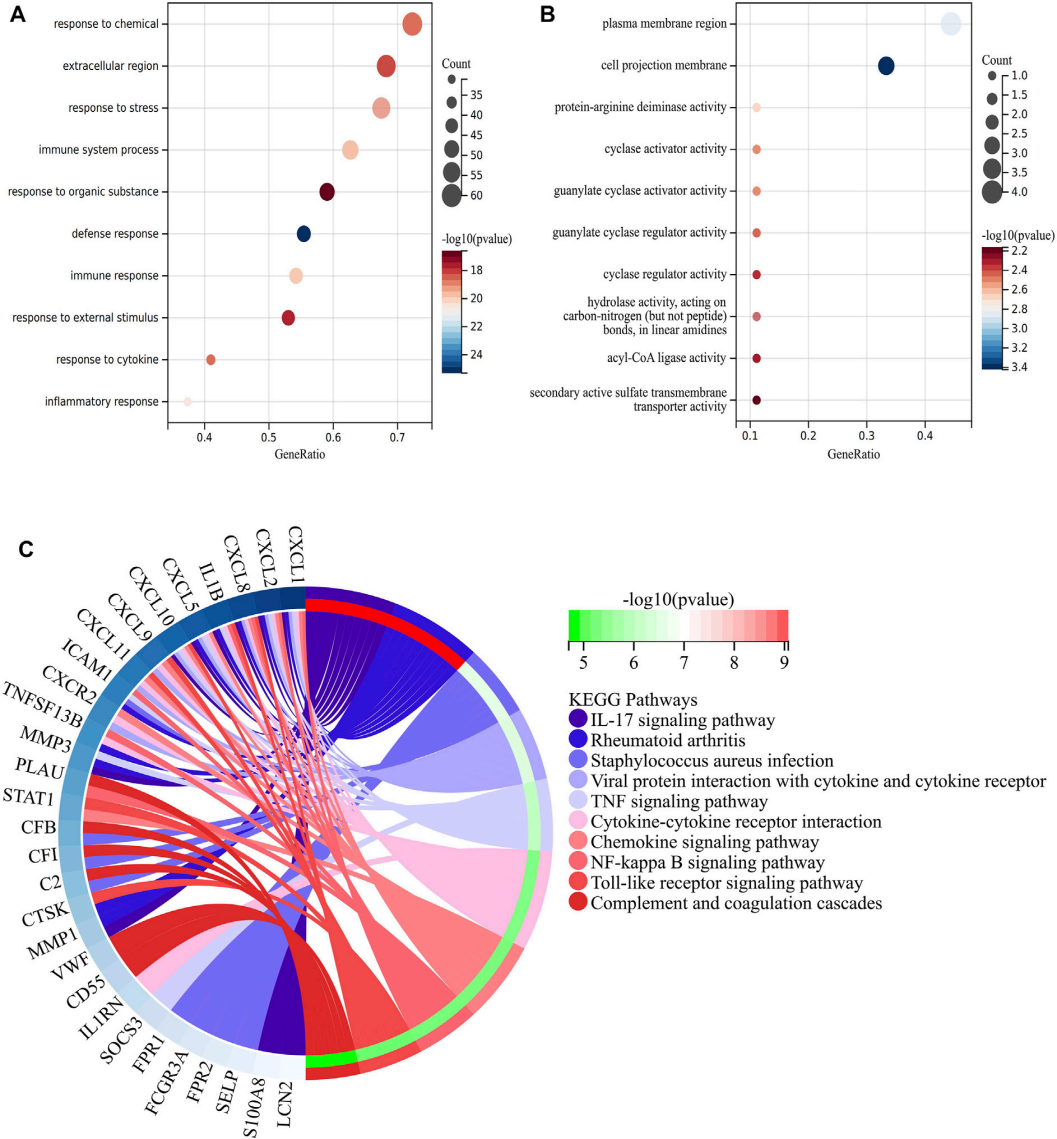
Function enrichment analysis of DEGs related to CD. **(A)** Bubble plot of enriched GO terms showing upregulated DEGs. **(B)** Bubble plot of enriched GO terms showing downregulated DEGs. A darker color and a larger bubble denote a more significant difference. **(C)** KEGG enrichment analysis of DEGs related to CD; The genes are linked to their assigned pathway terms via colored ribbons and are ordered according to the observed log10 *p*-value, which is displayed in descending intensity of red-green squares next to the selected genes.

In order to make predictions about the biological functions of DEGs, we carried out functional enrichment analysis of upregulated and downregulated genes. Results of GO analysis showed that the upregulated genes were mainly enriched in immune system process, response to stress, response to cytokine and extracellular region (Figure 3A), while the downregulated genes were significantly enriched in cell projection membrane and plasma membrane region (Figure 3B). KEGG pathway analysis indicated that the DEGs were significantly enriched in Cytokine-cytokine receptor interaction, IL-17 signaling pathway, Rheumatoid arthritis and TNF signaling pathway (Figure 3C).

### PPI network construction, module analysis and hub genes identification

**FIGURE 4:**
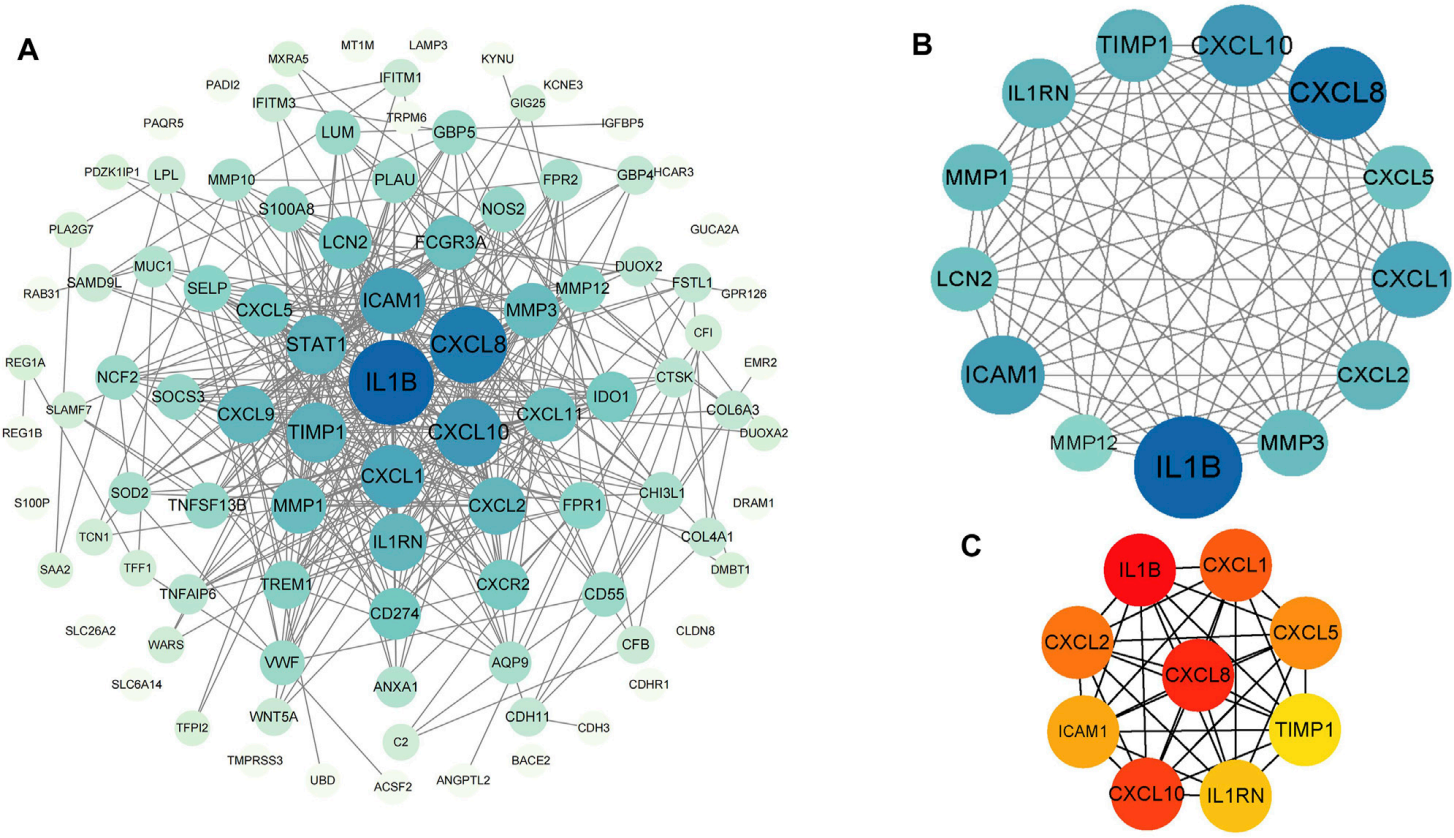
PPI networks of 88 upregulated genes and 9 downregulated genes by Cytoscape. The network consists of 97 nodes and 376 edges. 2 edges between nodes represent the interactions between genes. Each gene corresponding to the node is sized and colored according to the degree of interaction. The color grade indicates the change in the degree of each gene from high (blue) to low (white). The nearer the blue node, the higher the connection between the 2 nodes **(A)**. The densest connected region in the PPI network (13 nodes, 75 edges) was identified using MCODE **(B)**. Using the MCC algorithm in cytoHubba, 10 hub genes were identified in the densest connected regions. The scores are shown in red color. A darker color means a higher score **(C)**.

PPI analysis of the DEGs was based on the STRING database and the results were visualized using Cytoscape (Figure 4A). Using MCODE, a plug-in in Cytoscape, we identified the most densely connected regions (13 nodes, 75 edges) in the PPI network (Figure 4B). The top 10 genes, including IL1B, CXCL8, CXCL10, CXCL1, CXCL2, CXCL5, ICAM1, IL1RN, TIMP1 and MMP3, were obtained using MCC algorithm with Cytoscape’s plug-in cytoHubba (Figure 4C). The results showed that IL1B (Interleukin 1 Beta, score 4.21E+07) and CXCL8(C-X-C motif chemokine ligand 8, score 4.21E+07) were the most significant genes, followed by CXCL10(C-X-C motif chemokine ligand 10, score 4.20E+07), CXCL1(C-X-C motif chemokine ligand 1, score 4.19E+07), CXCL2(C-X-C motif chemokine ligand 2, score 4.19E+07), CXCL5(C-X-C motif chemokine ligand 5, score 4.18E+07), ICAM1(Intercellular adhesion molecule 1, score 4.17E+07), IL1RN(Interleukin-1 receptor antagonist protein, score 4.10E+07), TIMP1(Metallopeptidase inhibitor 1, score 4.03E+07) and MMP3(Matrix metalloproteinase-3, score 4.03E+07).

### Analysis of hub genes

**FIGURE 5:**
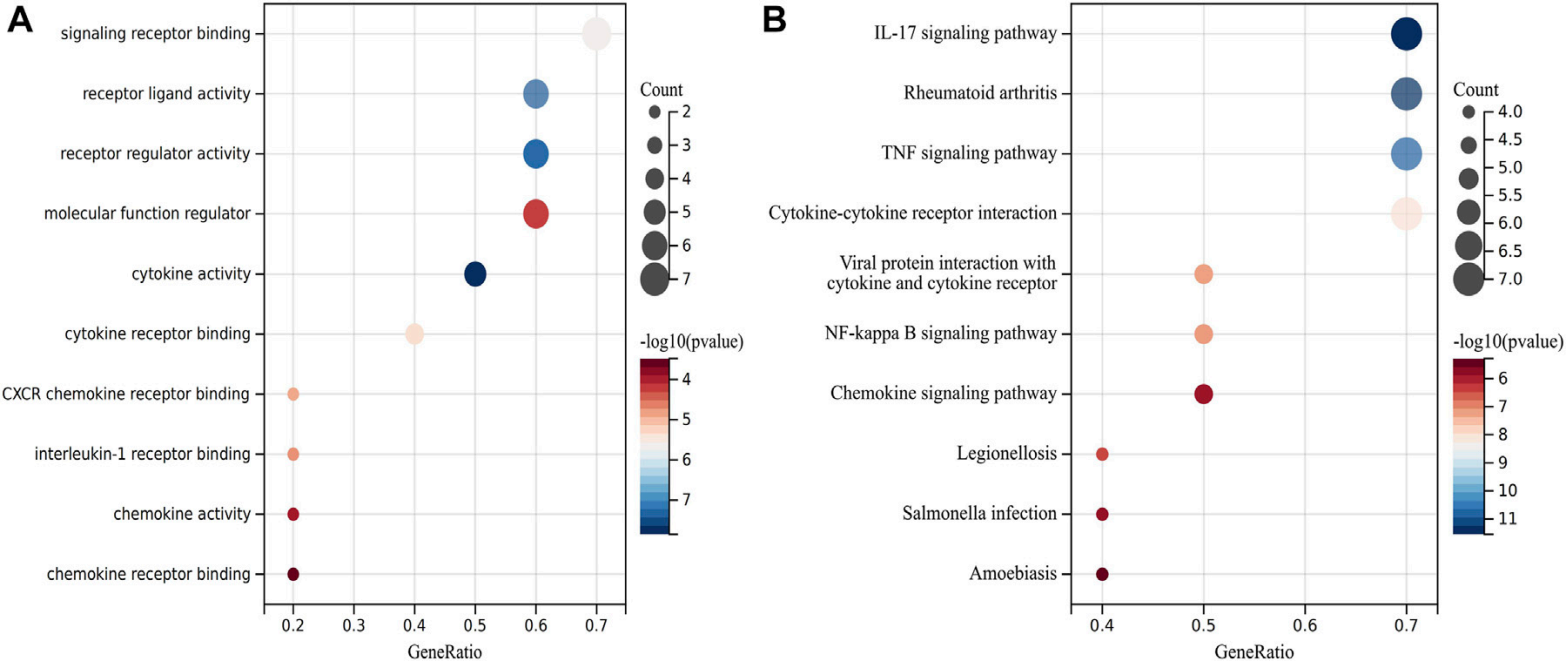
Analysis of functional enrichment for hub genes. **(A)** Bubble plot of enriched GO terms showing hub genes. **(B)** Bubble plot of enriched KEGG showing hub genes.

The symbols, abbreviations and functions of the hub genes are listed in Table 3. Functional enrichment analysis revealed 10 hub genes mainly centered on biological processes (BP), such as cytokine-mediated signaling pathway, regulation of signaling receptor activity, cellular response to cytokine stimulus, response to cytokine, while KEGG was mainly focused on IL-17 signaling pathway, Rheumatoid arthritis, TNF signaling pathway, Cytokine-cytokine receptor interaction and NF-kappa B signaling pathway (Figures 5A,B; Table 4).

**TABLE 3:** 10 hub genes and their functions.

Gene symbol	Description	Function
IL1B	Interleukin 1 Beta	Potent proinflammatory cytokine
CXCL8	C-X-C motif chemokine ligand 8 (IL-8)	A chemotactic factor that attracts neutrophils, basophils, and T-cells, but not monocytes
CXCL10	C-X-C motif chemokine ligand 10	Chemotactic for monocytes and T-lymphocytes. Binds to CXCR3
CXCL1	C-X-C motif chemokine ligand 1	Has chemotactic activity for neutrophils. May play a role in inflammation and exerts its effects on endothelial cells in an autocrine fashion
CXCL2	C-X-C motif chemokine ligand 2	Produced by activated monocytes and neutrophils and expressed at sites of inflammation
CXCL5	C-X-C motif chemokine ligand 5	Involved in neutrophil activation
ICAM1	Intercellular adhesion molecule 1	ICAM proteins are ligands for the leukocyte adhesion protein LFA-1
IL1RN	Interleukin-1 receptor antagonist protein	Inhibits the activity of interleukin-1 by binding to receptor IL1R1 and preventing its association with the coreceptor IL1RAP for signaling
TIMP1	Metallopeptidase inhibitor 1	Metalloproteinase inhibitor that functions by forming one to one complexes with target metalloproteinases
MMP3	Matrix metalloproteinase-3	Can degrade fibronectin, laminin, gelatins of type I, III, IV, and V; collagens III, IV, X, and IX, and cartilage proteoglycans

**TABLE 4:** Functional enrichment analysis of hub genes.

Term	Description	Count in gene set	P.value	Gene symbol
GO: BP	cytokine-mediated signaling pathway	9	4.30037448176226e-12	IL1B/CXCL8/CXCL10/CXCL1/CXCL2/ICAM1/IL1RN/TIMP1/MMP3
GO: BP	regulation of signaling receptor activity	8	5.47014566250108e-11	IL1B/CXCL8/CXCL10/CXCL1/CXCL2/CXCL5/IL1RN/TIMP1
GO: BP	cellular response to cytokine stimulus	9	9.99274690868016e-11	IL1B/CXCL8/CXCL10/CXCL1/CXCL2/ICAM1/IL1RN/TIMP1/MMP3
GO: BP	response to cytokine	9	1.93718003768327e-10	IL1B/CXCL8/CXCL10/CXCL1/CXCL2/ICAM1/IL1RN/TIMP1/MMP3
KEGG	IL-17 signaling pathway	7	2.87111108913105e-12	IL1B/CXCL8/CXCL10/CXCL1/CXCL2/CXCL5/MMP3
KEGG	Rheumatoid arthritis	7	2.87111108913105e-12	IL1B/CXCL8/CXCL1/CXCL2/CXCL5/ICAM1/MMP3
KEGG	TNF signaling pathway	7	1.09096029745403e-11	IL1B/CXCL10/CXCL1/CXCL2/CXCL5/ICAM1/MMP3
KEGG	Cytokine-cytokine receptor interaction	7	9.92447566156695e-09	IL1B/CXCL8/CXCL10/CXCL1/CXCL2/CXCL5/IL1RN
KEGG	Viral protein interaction with cytokine and cytokine receptor	5	6.98290442829672e-08	CXCL8/CXCL10/CXCL1/CXCL2/CXCL5
KEGG	NF-kappa B signaling pathway	5	7.71707732164516e-08	IL1B/CXCL8/CXCL1/CXCL2/ICAM1

### Validation of hub gene expression in CD

**FIGURE 6:**
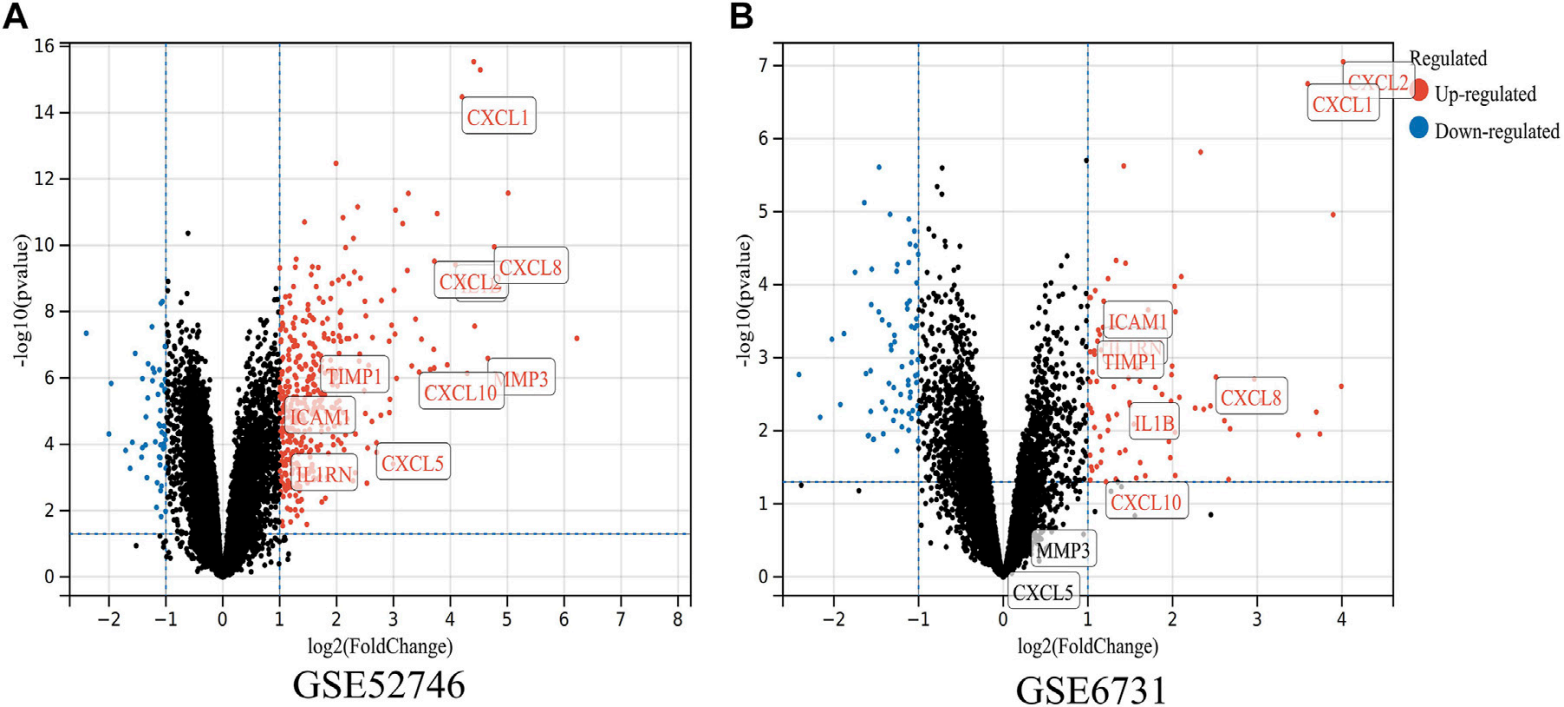
Validation of the expressions of hub genes in CD. **(A,B)** DEGs of the GSE52746 and GSE6731 datasets are shown, separately. Red data points represent upregulated genes and blue ones represent downregulated genes. Genes without any significant differences are in black.

To determine whether the hub genes were differentially expressed in the datasets of CD, we selected two other microarray datasets (GSE52746 and GSE6731) for analysis. A total of 464 DEGs were found in GSE52746 and 167 DEGs were found in GSE6731 (Figures 6A,B). In dataset GSE52746, all 10 previously screened hub genes were upregulated DEGs; whereas in dataset GSE6731, all the hub genes were upregulated DEGs except CXCL5 and MMP3.

### Establishment of miRNAs-hub genes regulatory network

**FIGURE 7:**
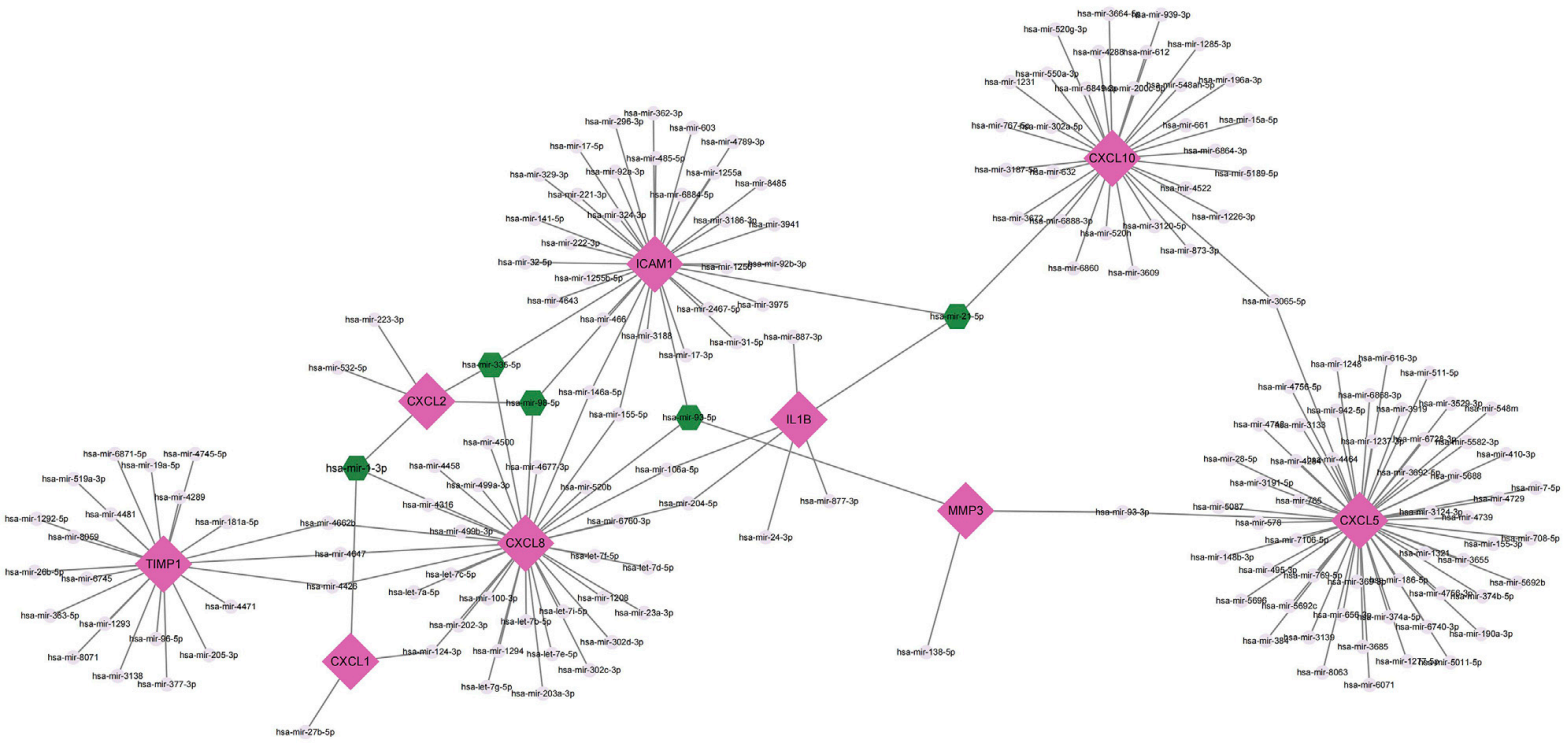
Top 9 hub genes in the integrated miRNA-DEGs network. The pink diamond shape indicates the 9 hub genes. The grey circles indicate miRNAs with low connective properties to the hub genes. Green hexagons indicate miRNAs with high connective properties to the hub genes.

MiRNAs perform multiple roles in regulating gene expression. Based on the NetworkAnalyst database, Cytoscape was used to construct miRNAs-hub genes regulatory networks to identify miRNAs aimed at hub genes. Finally, all of the 10 genes, with the exception of IL1RN, were identified to be related to miRNAs.9 hub genes and their correspondent regulatory miRNAs molecules are shown in Figure 7 and Table 5. Hsa-mir-21-5p had 3 target genes (ICAM1, CXCL10 and IL1B). Among the 9 hub genes, CXCL2, CXCL8 and ICAM1 were common targets of 2 miRNAs (hsa-mir-98-5p and hsa-mir-335-5p).

**TABLE 5:** The critical miRNAs in CD.

Name	Degree	Genes of interaction	Betweenness score
hsa-mir-21-5p	3	ICAM1, CXCL10, IL1B	4,301.029
hsa-mir-93-5p	3	ICAM1, MMP3, CXCL8	4,839.289
hsa-mir-98-5p	3	CXCL2, CXCL8, ICAM1	783.4889
hsa-mir-1-3p	3	CXCL1, CXCL2, CXCL8	275.1575
hsa-mir-335-5p	3	CXCL2, CXCL8, ICAM1	783.4889

## Discussion

Bioinformatics studies have enriched the field of complex polygenic diseases and have helped to identify several genes responsible for CD, thus providing new insights into the pathogenesis of CD. In this study, 97 DEGs were identified, consisting of 88 upregulated genes and 9 downregulated genes. The results of GO functional classification indicated that the DEGs were mainly enriched in immune system process, response to stress, response to cytokine and extracellular region. KEGG pathway analysis indicated that the DEGs were significantly enriched in IL-17 signaling pathway, Rheumatoid arthritis, TNF signaling pathway and Cytokine-cytokine receptor interaction. In the PPI network of DEGs, 10 (IL1B, CXCL8, CXCL10, CXCL1, CXCL2, CXCL5, ICAM1, IL1RN, TIMP1 and MMP3) out of 97 genes had high degree of interation. All of the 10 hub genes were upregulated in patients with CD. GO term analysis showed that these 10 genes were highly enriched in cytokine-mediated signaling pathway, regulation of signaling receptor activity, cellular response to cytokine stimulus, response to cytokine, while KEGG pathway analysis were mainly enriched IL-17 signaling pathway, Rheumatoid arthritis, TNF signaling pathway, Cytokine-cytokine receptor interaction and NF-kappa B signaling pathway. Numerous studies have suggested that the pro-inflammatory cytokine IL17 is associated with the pathogenesis of CD (Schmitt et al., 2021). Through the action of the TNF signaling pathway, anti-TNF therapy was approved for Crohn’s disease in 1998 and has transformed the treatment landscape, allowing for improved patient response and remission rates (Adegbola et al., 2018). A variety of complex roles regarding NF-κB signaling in the pathogenesis of IBD have also been elucidated in previous studies (Huang et al., 2019; Nguyen et al., 2021). These enrichment results for GO terms and the KEGG pathway indicate that the DEGs or hub genes found in our study might be participating in the disease progression of CD by the aforementioned means.

The IL17 and IL23 signaling pathways could trigger a cascade of pro-inflammatory molecules such as TNF, IL22, lymphotoxin, IL1B and lipopolysaccharide (LPS) thus affecting the progression of CD (Schmitt et al., 2021). IL23 binding to the receptor activates Janus kinase 2 (JAK2) and tyrosine kinase 2 (TYK2), which leads to subsequent signal transduction and phosphorylation of transcriptional activator 3 (STAT3) in the p19 subunit and STAT4 in the p40 subunit, and subsequent IL23R signaling initiation leads to the activation of several pathways including P38 MAPK, PI3K-Akt, and NFкB. Activation of these pathways leads to the release of CD-associated cytokines such as IL17A, IL17F, or IL22, which contribute in the pathogenesis of CD (Cho et al., 2006; Floss et al., 2013; Razawy et al., 2018).

IL1B (IL-1β) is a pivotal mediator in the inflammatory response and is essential for both host response and defense against pathogens (Lopez-Castejon and Brough, 2011). It has been shown that alterations in IL1B gene expression can be a predictive factor for non-response to anti-TNF treatment among patients with CD (Lykowska-Szuber et al., 2021). It has been indicated that IL-1β could be a target for potential clinical intervention in patients with colitis who have not responded to the neutralization of TNFα (De Santis et al., 2017). In the present study, IL1B was the most significant upregulated gene, which indicated its possible use as a potential indicator for the diagnosis of CD.

CXC chemokines can be divided into two groups: the ELR + CXC family is structurally characterized by a Glu-Leu-Arg tripeptide pattern at its N-terminal end; CXCL1, CXCL2, CXCL5, and CXCL8 belong to the ELR + CXC family. Unlike the ELR + CXC family, the ELR-CXC family lacks this tripeptide pattern, to which CXCL10 belongs (Clark-Lewis et al., 1993; Strieter et al., 1995). Several ELR + CXC chemokines have been identified in association with IBD: CXCL1-2, CXCL5 and CXCL8 chemokines are significantly expressed in areas of intestinal inflammation in patients with IBD compared to normal tissues (Autschbach et al., 2002; Banks et al., 2003; Gijsbers et al., 2004). Dhawan et al. showed that high CXCL8 expression was associated with reduced expression of choline acetyltransferase in resected intestinal epithelial cells from patients with CD (Dhawan et al., 2015). ELR-CXC chemokines are highly responsive to memory T cells and NK cells (Cole et al., 1998; Cole et al., 2001). CXCL10 is a ligand for the CXCR3 receptor and its activation leads to the recruitment of T lymphocytes and the perpetuation of mucosal inflammation (Ostvik et al., 2013). It has been suggested that atorvastatin to reduce plasma CXCL10 levels may be a candidate for future treatment of Crohn’s disease (Grip and Janciauskiene, 2009). In our study, all 5 chemokines were upregulated in CD patients, suggesting a potential role in the future as biological targets to forecast and guide CD therapy.

ICAM1 causes leukocytes to migrate to the inflamed mucosa by binding to its receptor. (Dustin et al., 1986). Anti-ICAM-1 antibodies have been shown to reduce colitis and prolong the survival of dss-induced ICAM-1-deficient mice (Bendjelloul et al., 2000). ICAM1 has been suggested as a possible early predictor that can determine the response to vedolizumab treatment in CD patients (Holmer et al., 2020). In combination with our study, ICAM1 may serve as a molecular target for the treatment of CD in the future.

TIMP1 is one of the four members of the glycoproteome (TIMP1-4), whose main function is the translocation of the extracellular matrix, while it is involved in various pathological processes, including wound healing (Gardner and Ghorpade, 2003). TIMP1 has been used as a predictor of CD-associated intestinal strictures (Zorzi et al., 2012). Further research is needed to determine whether TIMP1 can be used as a therapeutic target for CD.

IL1RN (IL-1RA) is a competitive inhibitor of naturally occurring interleukin-1 (IL-1)-induced pro-inflammatory activity (Witkin et al., 2002). Dobre et al. suggested that transcript levels of IL1RN are candidate biomarkers that can contribute to the differential diagnosis of UC and CD in clinical practice (Dobre et al., 2018). A study by Bank et al. suggested that genetic polymorphisms involved in the regulation of the cytokine pathway (IL1RN) were associated with the response to anti-TNF therapy (Bank et al., 2019). Infliximab is effective in inducing and maintaining remission in CD patients, and MMP3 has been shown to be a promising biomarker for predicting primary non-response to infliximab (Li et al., 2021). The role of MMP3 and IL1RN in CD is still unexplored and more studies are needed to clarify it.

For microRNAs (miRNAs), a major role is to regulate the expression of most human genes; they perform a crucial function in the development of autoimmune diseases, including CD (Zhou et al., 2021a). The results of our study suggest that several miRNAs, including hsa-mir-21-5p, hsa-mir-93-5p, hsa-mir-98-5p, hsa-mir-1-3p, and hsa-mir-335-5p, may play critical roles in CD. It has been shown that elevated levels of miR-21-5p in the stool of IBD patients could be a guide for the noninvasive clinical diagnosis of IBD (Zhou et al., 2021b). It has been demonstrated that miR-93-5p is upregulated before surgery and downregulated in relapsed CD patients (Moret-Tatay et al., 2021). Wang et al. found that the lncRNA MEG3 could improve ulcerative colitis by upregulating miR-98-5p-Sponed IL-10 (Wang et al., 2021). It has been shown that MiR-1-3p and MiR-124-3p could synergistically disrupt the intestinal barrier in the aging colon to promote the development of IBD (Sun et al., 2022). However, the relationship between mir-335-5p and CD has not been reported and needs to be explored further. In previous studies, mir-335-5p has been found to inhibit the inflammatory response in chronic rhinosinusitis (Gu et al., 2020); and moreover mir-335-5p could alleviate the inflammatory response and airway fibrosis by modulating ATG5, resulting in relief of childhood asthma (Liang et al., 2022). It is also shown that fibroblasts with high ICAM1 expression act as a key driver of inflammation and play a facilitative role in the process of fibrosis (Layton et al., 2020). In our study, miR-335-5p was interlinked with CXCL2, CXCL8 and ICAM1, which led us to speculate that miR-335-5p may alleviate the progression of CD by suppressing the intestinal inflammatory response (CXCL2, CXCL8) and intestinal fibrosis (ICAM1). These results may provide us with new research ideas about their interactions in CD. In addition, studies about genes and miRNAs in CD remains to be limited.

There is no doubt that gene-miRNA regulatory networks act as an essential role in the CD mechanism. This not only enhances the understanding of CD, but also provides targeted therapeutic strategies and predictions for CD. The study is limited in that microarray expression profiles were analyzed using bioinformatics analysis and not validated with primary experiments. Additionally, we did not explore the detailed mechanisms for how hub genes and miRNAs modulate CD. As a result, further validation of our findings with additional clinical samples and research is necessary in the future.

## Conclusion

In conclusion, a total of 97 DEGs, 10 hub genes and 5 miRNAs (hsa-mir-21-5p, hsa-mir-93-5p, hsa-mir-98-5p, hsa-mir-1-3p, and hsa-mir-335-5p) that may be involved in the progression or occurrence of CD were identified in this study, which could be regarded as biomarkers of CD. In addition, these hub genes act mainly on IL-17 signaling pathway, TNF signaling pathway, and NF-kappa B signaling pathway to influence the progression of CD. However, further studies are still needed to define their biofunction in CD.

## Data Availability

Publicly available datasets were analyzed in this study. This data can be found here: https://www.ncbi.nlm.nih.gov/geo/.
